# Acute management of ocular chemical injury using a combination eyelid retraction and irrigation instrument

**DOI:** 10.1016/j.aopr.2021.100003

**Published:** 2021-09-03

**Authors:** William R. Bloom, Jay P. Mathias, Srinivas Sai A. Kondapalli

**Affiliations:** Chicago Medical School, Rosalind Franklin University of Medicine and Science, 3333, Green Bay Rd, North Chicago, IL, USA; Ohio University Heritage College of Osteopathic Medicine, 191 W Union St, Athens, OH, USA; Everett and Hurite Ophthalmic Association, 1835, Forbes Avenue, Pittsburgh, PA, USA

Dear Editor,

Accounting for up to 36,000 emergency department visits annually in the United States, chemical ocular burns are a common ophthalmic injury presenting to emergency and urgent care settings.^1^ Exposure to acids and alkalis can cause serious and irreversible ocular morbidity.^2^ Early intervention may limit tissue damage and preserve vision. Management recommendations include prompt irrigation until the conjunctival fornix pH returns to physiologic levels and continued monitoring of pH once stable.^2^^,^^3^ Eyelid retraction and eversion is suggested to eliminate offending agents in recessed ocular tissues (the conjunctival fornix and palpebral conjunctiva) and to flush out potentially embedded foreign bodies.

Irrigation following chemical injury may be complicated by patient cooperation and user skill.^4^^,^^5^ Current available devices used for ocular irrigation have their limitations. For instance, a large volume syringe is often the only available irrigation device after chemical exposure on the front line.^3^^,^^6^ This alone does not facilitate the eyelid retraction necessary to confidently irrigate the conjunctival fornices and palpebral conjunctiva. Residual chemicals may prolong exposure and increase the risk of severe ocular injury and visual disability.^5^^,^^9^^,^^10^ While effective techniques and timely irrigation mitigates the potential for permanent damage after chemical exposure, it is clear there are unmet needs in the management of ocular burns***.***

Here we introduce a combination eyelid retractor and irrigation system and describe a pilot study comparing this instrument with currently used early irrigation practices for chemical injury using a porcine model. The eyelid retractor and irrigation instrument (Fig. 1), attaches directly to a standard 10-cc syringe via Luer taper and has been used previously in humans without complications to irrigate the eye after an in-office ophthalmologic procedure.^7^ The instrument allows the user to retract eyelid while simultaneously irrigating the conjunctival fornix, palpebral and bulbar conjunctiva, and corneal surface.

**Fig. 1:**
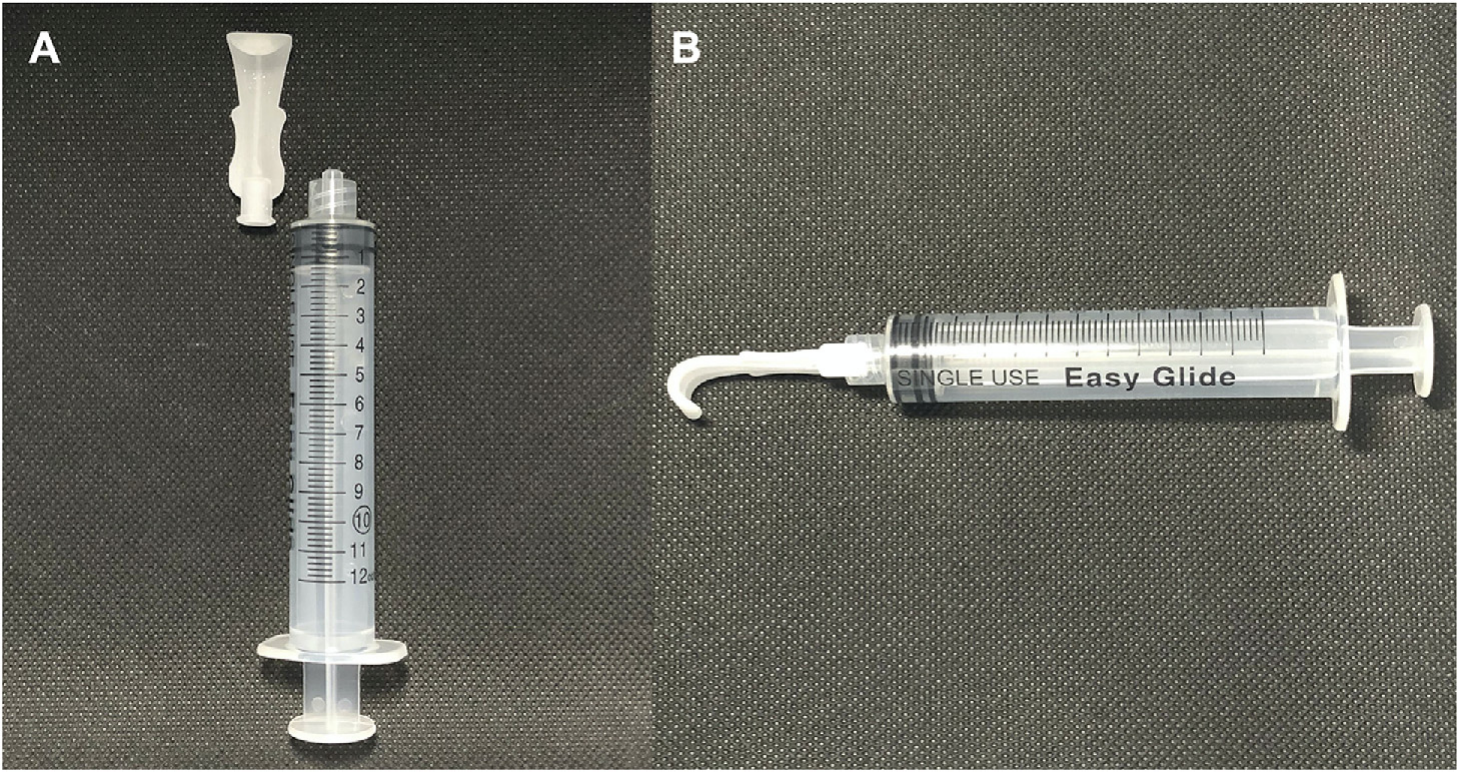
(A) Eyelid retractor instrument with irrigation ports and a 10-cc syringe. (B) Instrument connected to a 10-cc syringe via Luer taper.

Given the nature of these injuries, an animal model was deemed appropriate for this initial investigation. Porcine models have shown to be a reliable tissue source to understand ocular injury and anatomy.^8^ Four whole porcine heads were included, allowing for simulated chemical injury and irrigation in eight eye and lid complexes. This study was performed in accordance with the Declaration of Helsinki's principles regarding animal use in biomedical research and no animals were sacrificed for the purposes of this research.

In this experiment, eyes were exposed to either an alkali or acid to simulate ocular chemical injury. Alkali chemical eye injury included four eyes receiving 1.0 mL of 10.0% sodium hypochlorite solution (13.0 pH). The solution was left in each eye for 1 ​min and the eyelids were mechanically moved in a blinking fashion ten total times. Simulation of acidic chemical eye injury included four eyes receiving 1.0 mL of 10.0% acetic acid solution (2.5 pH). Again, the solution was left in each eye for 1 ​min and the eyelids were mechanically moved in a blinking fashion ten total times.

To assess standard chemical eye injury management, two eyes in the simulated alkali injury and two eyes in the simulated acid injury were rinsed with saline directly to the eye and eyelid complex via a 10-cc syringe. This process was performed three times, allowing for a final irrigation with 30-cc of saline. Next, two eyes in the simulated alkali injury and two eyes in the simulated acid injury were rinsed with the eyelid retractor with irrigation ports. The instrument was connected to a saline filled 10-cc syringe and was used to retract the upper eyelid (Fig. 2) with subsequent depression of the syringe plunger to simultaneously irrigate the eye. This process was repeated two additional times to allow for irrigation with 30-cc of saline. One minute after each rinse procedure, the pH was measured with a hand-held digital pH meter (GAIMC, Shaanxi, China).

**Fig. 2:**
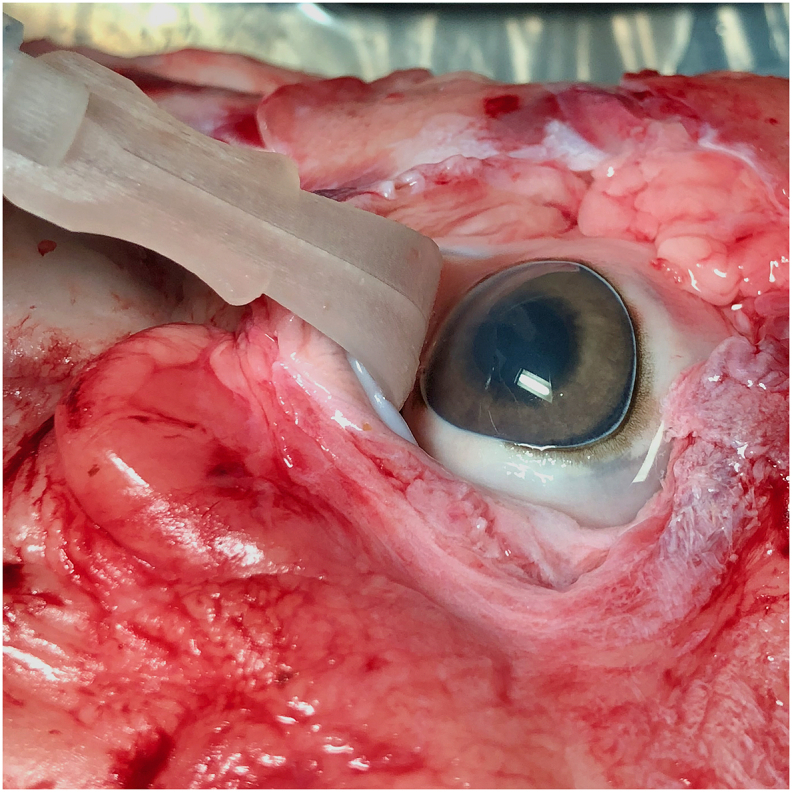
Demonstration of the instrument in a porcine model with retraction of the upper eyelid.

A single pH measurement of 7.2 was collected from one porcine eye to establish an approximate baseline tear film pH. For the purposes of this report, this measurement was considered the pre-rinse pH standard. The pH was recorded after simulated acid or alkali injury and irrigation using either a 10-cc syringe alone or the combination eyelid retractor and irrigation system. The mean ​± ​SD post-rinse pH in eyes exposed to sodium hypochlorite and irrigated with 30 mL of saline using a 10-cc syringe alone was 10.2 ​± ​0.15. The mean ​± ​SD post-rinse pH in eyes exposed to sodium hypochlorite and irrigated with 30 mL of saline using the combination eyelid retractor and irrigation instrument was 9.4 ​± ​0.15. The mean ​± ​SD post-rinse pH in eyes exposed to acetic acid and irrigated with 30 mL of saline using a 10-cc syringe alone was 3.5 ​± ​0.05. The mean ​± ​SD post-rinse pH in eyes exposed to acetic acid and irrigated with 30 mL of saline using the combination eyelid retractor and irrigation instrument was 5.0 ​± ​0.05.

With the same total volume of saline, irrigation using the novel device resulted in a non-inferior conjunctival fornix pH improvement compared to irrigation with a 10-cc syringe alone. The findings in this report suggest an eyelid retractor and irrigation system may even reduce the chemical load in the conjunctiva and fornix following immediate irrigation.

Timely management after exposure to acid or alkali agents may mitigate the risk of tissue damage. This includes immediate irrigation of the eye and its surrounding structures.^9^ Incomplete irrigation prolongs exposure and exacerbates ocular damage following chemical injury.^10^ There continues to be a need for an easy-to-use system that provides a thorough irrigation after chemical exposure in the absence of a permanent eyewash station. This single-use, easily transportable device may be used as a first-aid prehospital intervention, as well as in urgent care, emergency department, or outpatient settings, to provide prompt irrigation with minimal training.

This study had several limitations, which should be considered when evaluating the findings. First, the sample sizes used in this pilot study were too small to appropriately employ statistical hypothesis testing. Second, the use of a porcine model limited our ability to draw conclusions using the device of interest in humans. Third, an experienced ophthalmologist performed all irrigation trials. Therefore, the results may differ when irrigation is performed by a layperson or first responder.

## Conclusions

The combination eyelid retractor and irrigation device appeared to be non-inferior to standard early intervention emergency irrigation practices used in ocular chemical injuries. By combining existing technology to improve irrigation practices, an eyelid retractor and irrigation system is unlikely to introduce additional risks not already associated with standard practices. Further research is necessary to address the aforementioned limitations and to confirm device efficacy compared to the current standards in the early management of chemical ocular burns.

## Study Approval

This study was performed in accordance with the Declaration of Helsinki's principles regarding animal use in biomedical research and no animals were sacrificed for the purposes of this research.

## Author Contributions

The authors confirm contribution to the paper as follows: Conception and design of study: SK, WB; Data collection: SK; Analysis and interpretation of results: SK, WB, JM; Drafting the manuscript: SK, WB, JM; All authors reviewed the results and approved the final version of the manuscript.

## Acknowledgments

Thanks to all the peer reviewers for their opinions and suggestions.

## Funding

This research did not receive any specific grant from funding agencies in the public, commercial, or not-for-profit sectors.

## Conflict of Interest

The authors declare the following financial interests/personal relationships which may be considered as potential competing interests: Dr. Kondapalli currently has a patent pending in the United States Patent and Trademark Office on the device described in this article. None of the other authors has a financial or proprietary interest in any method or material mentioned in this report and no financial support was used for the completion of this manuscript.
